# China’s little-known efforts to protect its marine ecosystems safeguard some habitats but omit others

**DOI:** 10.1126/sciadv.abj1569

**Published:** 2021-11-12

**Authors:** John J. Bohorquez, Guifang Xue, Timothy Frankstone, Maria M. Grima, Karine Kleinhaus, Yiyi Zhao, Ellen K. Pikitch

**Affiliations:** 1 Stony Brook University, School of Marine and Atmospheric Sciences, Stony Brook, NY 11794, USA.; 2 Institute for Ocean Conservation Science, Stony Brook University, Stony Brook, NY, 11794 USA.; 3 KoGuan Law School, Shanghai Jiao Tong University, Shanghai, China.; 4 School of Global Public Health (GPH), New York University, New York, NY 10012, USA.

## Abstract

China’s stature as the world’s major producer and consumer of seafood is legendary, but its long-standing tradition of protecting marine life domestically is virtually unknown. We present the most comprehensive database on area-based marine conservation in China including 326 sites that conserve 12.98% of China’s seas and address 142 conservation objectives. Twenty-two percent of shallow habitats (<10 meters) were fully or highly protected and 20% of waters 10 to 50 meters deep were conserved to some degree. Ecosystems in deeper waters (>50 meters) are critical to protect, yet <5% of these waters in China were conserved, primarily in areas with the highest chlorophyll-α concentrations. Habitats such as underwater canyons and seamounts beyond the continental shelf had no area-based protection. While China has made progress in marine protection within its boundaries, there is more work to be done to ensure that the full suite of marine life is safeguarded.

## Teaser

China has made substantial progress towards global targets for conserving coastal habitats but less headway for deeper waters.

## INTRODUCTION

The People’s Republic of China is the largest producer and consumer of life in the ocean, both farmed and wild-caught (*1*–*3*). Its fleets haul from domestic seas, foreign territories, and waters beyond any national jurisdiction, with 39% of the country’s wild-caught fisheries estimated to come from abroad (*4*). Recent international news headlines have highlighted China’s large-scale—and sometimes illegal—fishing in or near other countries, with examples in South America, North Korea, and West Africa in 2020 and 2021 (*5*–*7*). It may therefore come as a surprise to experts and nonexperts alike that China has a long-standing tradition of strong marine conservation within its own seas and coastal habitats.

Marine protected areas (MPAs) have been in place in China for nearly six decades. From national parks to fishery reserves, MPAs include a wide variety of measures that are expressly intended to conserve biodiversity and provide comprehensive protection for all life within their borders (*8*, *9*). MPAs are rising in popularity around the world because of their potential to preserve and restore marine ecosystems, enhance their resilience to climate change impacts, and for the socioeconomic benefits that can result from healthy marine environments (*10*–*12*). Global policies for marine conservation have also recently recognized other effective area-based conservation measures (OECMs) as spatial zones that may not meet all the criteria for MPAs but still achieve comparative protection for biodiversity and can count toward goals for protected areas. While the global community has yet to agree upon a strict definition for OECMs, fishery management zones have particularly been referenced as potential candidates (*13*), some of which are also long-standing traditions in China.

Mainland China has an extensive network of MPAs, with several established in every coastal province. They address a wide range of conservation objectives, providing protection for species and ecosystems of high ecological, cultural, and economic value. MPAs and other area-based conservation measures in China (*14*) include:

### Marine nature reserves

These MPAs protect rare or endangered species and ecosystems. They are no-take, meaning any extraction of fish or other living resources is illegal. Ecotourism and scientific research are allowed in “buffer zones” that surround the “core zone” where only staff are permitted (*15*, *16*).

### Special marine protected areas

Special MPAs focus on sustainable use of sensitive or rare natural resources, including biodiversity and nonliving geological features. Special MPAs allow for research, aquaculture (farming), ecotourism, and some sustainable fishing. While not fully no-take, special MPAs can have no-take core zones. Marine parks are a subcategory of special MPAs that have a particular focus on ecotourism. Special MPAs are sometimes known as marine special protected areas (*15*, *16*).

### Aquatic germplasm reserves

Aquatic germplasm reserves (AGRs), also known as Fishery Conservation Zones, are conservation areas that protect commercially important, rare, or endangered fish species. They frequently target important reproductive areas including breeding and nursery grounds, as well as known migration routes. Unlike MPAs, the primary objective of AGRs is not biodiversity conservation, as required of MPAs by the International Union for the Conservation of Nature. However, prior research indicates that AGRs may qualify as OECMs because comprehensive biodiversity conservation may be an outcome of AGRs and thus may align with MPA goals (*13*, *14*).

Ecological red line areas along China’s coastline are also important tools for marine spatial planning in China but with a focus on restricting development and improving water quality rather than fishing and other related activities for biodiversity conservation and protection (*17*). As a result, MPAs and AGRs are often coordinated with and overlap red line areas, but red-line areas would not qualify as MPAs or OECMs and would lead to double counting area protected. Therefore, red line areas were not directly incorporated into this analysis, although many of the MPAs and AGRs in this research are part of red line systems.

Despite China’s global political and environmental relevance, little is known about the 270+ MPAs reported to exist in the country (*18*–*20*). For example, as of October 2021, the United Nations (UN)–officiated World Database on Protected Areas (www.protectedplanet.net; accessed 14 October 2021) only contained 15 Chinese MPAs in its dataset.

Globally agreed targets historically called for protecting 10% of the ocean by the year 2020 (*21*–*23*). Some agreements emphasize that the targets should be achieved in an “ecologically representative” manner whereby protection is extended across the diversity of life in the ocean (*24*). Experts are concerned that in practice, there has been greater focus on the quantity of area protected than in its distribution or quality. This may result in uneven representation of important habitats with a relatively small number of specific biogeographical regions well conserved (*25*–*27*).

Targets for the years 2021–2030 are being discussed as policy decision makers, scientific experts, and other stakeholders from around the world convene at the Convention on Biological Diversity’s Conference of the Parties, which will conclude in Kunming, China in 2022. As of September 2021, over 100 countries had publicly supported a goal to protect 30% of the global ocean by 2030 (*28*). China’s global relevance to marine biodiversity, the lack of information on conservation in the country, and the upcoming high-level meeting in China, all combine to make analysis and dissemination of information on Chinese marine conservation especially timely, pressing, and of unusually high global importance.

Our collaborative United States and China–based research team developed the following three key objectives to advance knowledge of area-based marine conservation in China:

1) Develop the first comprehensive, publicly available database [see auxiliary database (see “Data and materials availability”)] of China’s area-based conservation measures.

2) Assess the distribution of China’s protected areas across different habitats as a measure of ecological representativeness.

3) Analyze China’s progress toward meeting internationally agreed conservation targets and pathways for improvement based on our study results.

The spatial scope of the analysis was China’s EEZ as determined by the UN Convention on Law of the Sea (UNCLOS) (*29*). However, all MPAs claimed by China, including those outside the UNCLOS-defined EEZ, are included in the database provided (see “Data and materials availability”).

## RESULTS

### Defined marine habitats in China

**Fig. 1.**
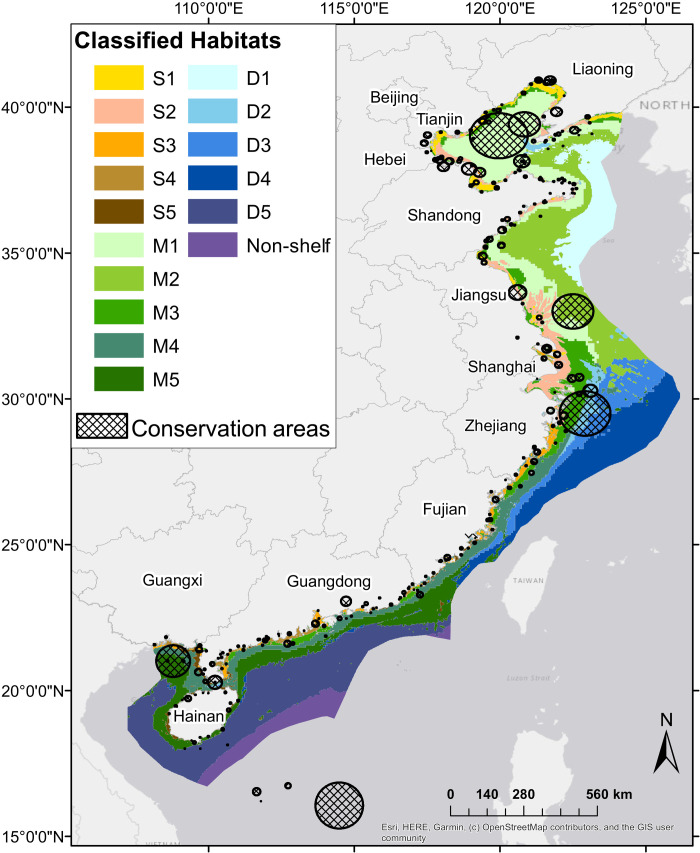
MPAs and AGRs overlapped with classified habitats. Classified habitats are limited to China’s UNCLOS-defined EEZ using materials from Esri and the Flanders Marine Institute (*49*). Map created in ArcMap 10.7.1 (*50*) using China Geodetic Coordinate System 2000. Provincial boundaries provided by ArcGIS Online via ArcMap data portal (table S2). Figure S1 depicts distribution of each classified habitat independently.

**Fig. 2.**
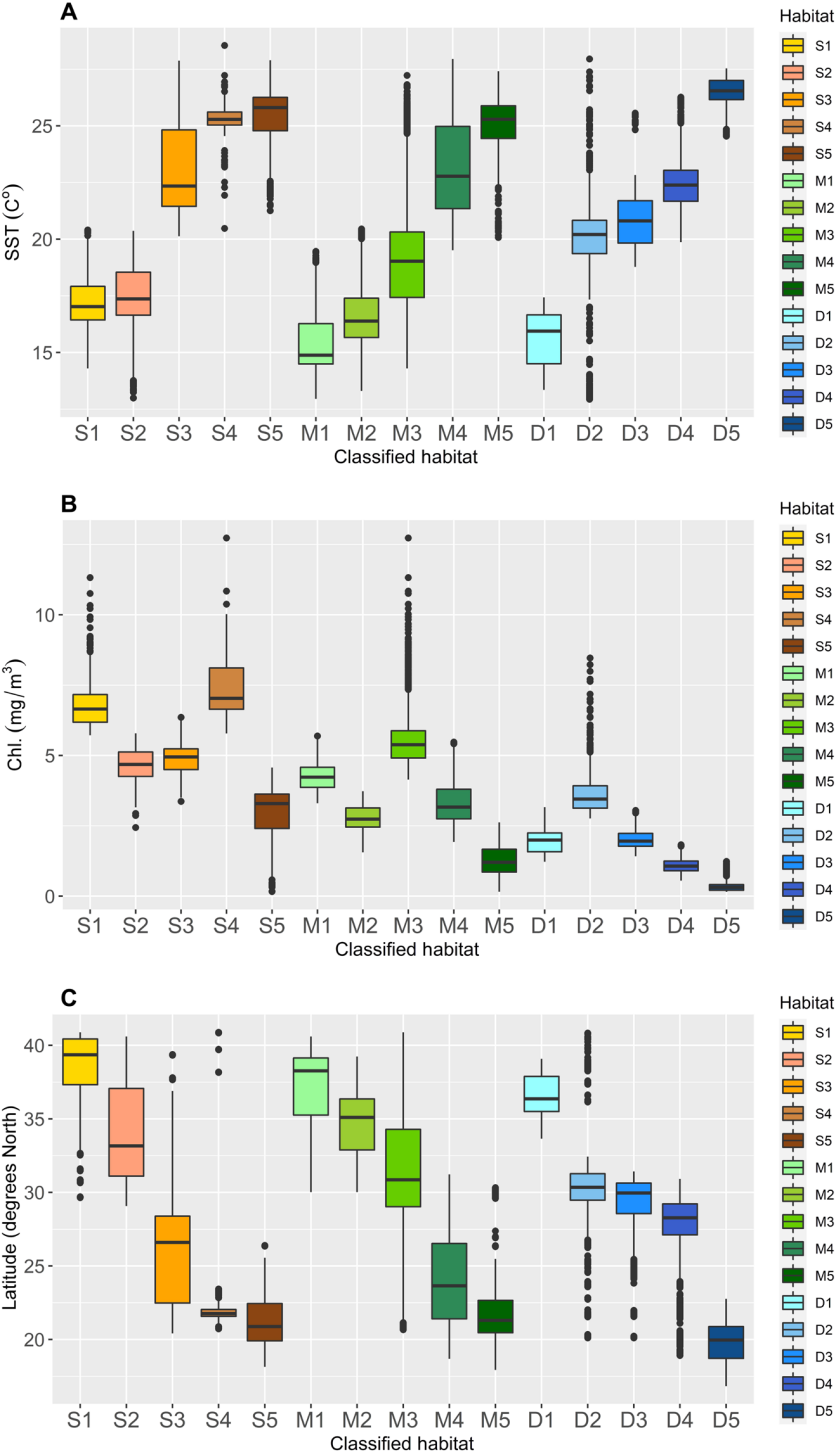
Box plots of characteristic features for classified habitats including (**A**) average annual sea surface temperature, (**B**) average annual chlorophyll-α concentration, and (**C**) latitude. Latitude was not used to inform the habitats directly but is depicted in this figure to visually display the distribution of the habitats north to south along China’s coastline. Chl., chlorophyll.

We classified 16 statistically distinct habitats in China from which to assess ecological representativeness of MPAs and AGRs throughout the country’s marine and coastal ecosystems (Figs. 1 and 2 and Table 1). Because detailed data on the basis of in situ measurements or observations were not available on a nationwide basis, we used remote sensing data to define habitats. Information used was a combination of satellite data on sea surface conditions (temperature and chlorophyll concentrations) and bathymetry from a database previously used to evaluate ecological representativeness of MPA networks in other countries (*26*, *30*, *31*). The 16 habitats were organized across three different depth zones relevant to marine life: the shallow depth zone (<10 m), medium depth zone (10 to 50 m), and deep depth zone (>50 m). We use alphanumeric codes to refer to the habitats that reflect their depth zone and relative position from north to south along China’s coastline according to their median latitudes (Fig. 2), such that habitat S1 is the northernmost habitat in the shallow depth zone and M5 is the southernmost habitat in the medium depth zone. Waters beyond the continental shelf were grouped into one habitat, referred to as “non-shelf,” that comprised less than 4% of the full extent of marine and coastal ecosystems in the study.

**Table 1.** Descriptive statistics of classified habitats from *k*-means analyses. Habitats initiating with “S-,” “M-,” and “D-” represent shallow, medium, and deep habitats, respectively. Chl.-α, chlorophyll-α. N/A, not applicable.

**Color**	**Habitat**	**Description**	**Habitat area** **(km^2^)**	**% of marine** **and coastal** **habitats**	**Mean annual** **SST (°C)**	**Mean annual** **Chl.-α** **(mg/m^3^)**	**Mean latitude** **(degrees)**	**Mean depth** **(m)**
	S1	Temperate, high productivity	15,406	1.77%	17.3 ± 1.09	6.80 ± 0.85	38.50 ± 2.39	5.0 ± 2.41
	S2	Temperate, moderate productivity	35,423	4.07%	17.5 ± 1.27	4.69 ± 0.54	34.0 ± 3.17	5.8 ± 2.39
	S3	Subtropical and tropical, moderate productivity	12,353	1.42%	22.9 ± 1.88	4.91 ± 0.53	25.9 ± 3.56	4.9 ± 2.47
	S4	Tropical, high productivity	4,173	0.48%	25.3 ± 0.76	7.42 ± 1.14	22.1 ± 2.40	4.5 ± 2.34
	S5	Tropical, low productivity	6,561	0.75%	25.4 ± 1.44	2.96 ± 0.96	21.1 ± 1.91	5.4 ± 2.46
	M1	Temperate, high productivity	124,099	14.27%	15.3 ± 1.18	4.25 ± 0.46	37.3 ± 2.48	21.3 ± 7.07
	M2	Temperate, moderate productivity, deeper	113,666	13.07%	16.4 ± 1.55	2.79 ± 0.43	34.9 ± 2.36	35.6 ± 9.46
	M3	Temperate to subtropical, very high productivity	54,740	6.30%	19.2 ± 2.52	5.56 ± 0.95	31.5 ± 5.19	20.7 ± 10.30
	M4	Tropical/ subtropical, moderate productivity	57,784	6.65%	23.2 ± 1.92	3.29 ± 0.68	24.1 ± 2.93	27.9 ± 11.40
	M5	Tropical/ subtropical, low productivity	67,511	7.76%	25.1 ± 1.32	1.28 ± 0.52	21.5 ± 1.78	35.5 ± 9.86
	D1	Temperate, moderate productivity	57,658	6.63%	15.7 ± 1.06	1.94 ± 0.39	36.6 ± 1.34	63.8 ± 8.67
	D2	Mixed temperatures, mostly subtropical, very high productivity	14,700	1.69%	19.3 ± 2.90	3.62 ± 0.66	31.3 ± 3.89	57.9 ± 7.87
	D3	Subtropical, moderate productivity	46,364	5.33%	20.8 ± 1.09	2.02 ± 0.33	29.3 ± 1.79	61.0 ± 7.33
	D4	Subtropical, low productivity	87,586	10.07%	22.3 ± 1.06	1.07 ± 0.24	27.9 ± 1.88	80.4 ± 14.00
	D5	Tropical, very low productivity	137,677	15.84%	26.5 ± 0.52	0.35 ± 0.18	19.8 ± 1.40	95.1 ± 36.60
	Beyond shelf	Habitats beyond the continental shelf	33,726	3.88%	N/A	N/A	N/A	N/A

### Ecological representation of MPAs and AGRs

**Fig. 3.**
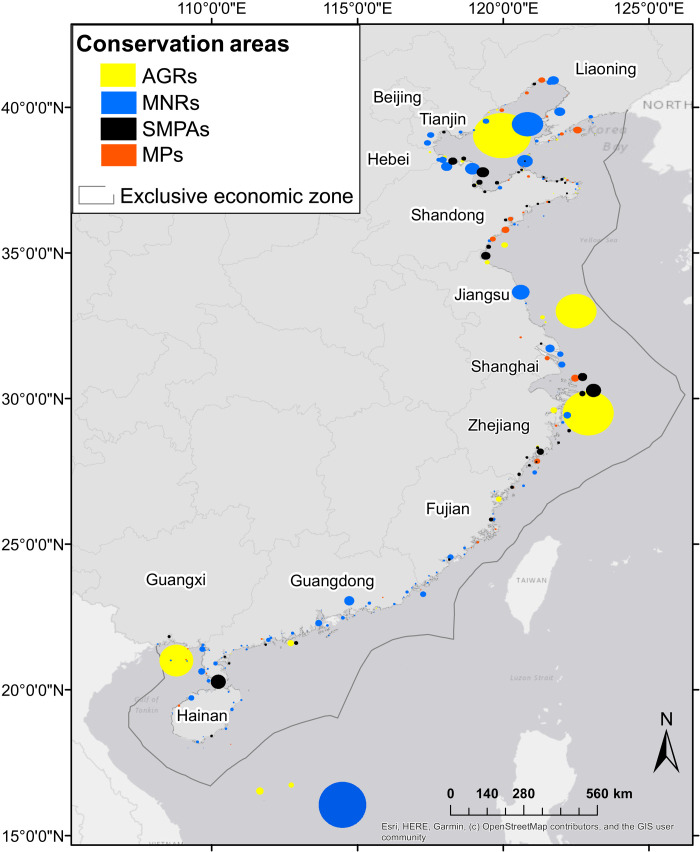
Spatial distribution of conservation areas by type: AGRs versus MPAs. Size of conservation areas correspond with known spatial coverage with spatial extent estimated as circles (see Materials and Methods). MPAs are represented across MNRs, SMPAs, and MPs as a subtype of SMPA. Names indicate coastal Chinese provinces. Map created in ArcMap 10.7.1 (*50*) using China Geodetic Coordinate System 2000. Provincial boundaries provided by ArcGIS Online via ArcMap data portal (table S2).

Our extensive review of China-based resources combined with expert consultations yielded a database of 273 MPAs and 53 AGRs. All MPAs have been fully implemented (see auxiliary data for year of establishment). Of these, limited data on 24 MPAs precluded their use in the analysis. Because these limited data areas were small municipal or locally managed MPAs (see the “Study limitations” section in Discussion) their exclusion is unlikely to have significantly affected results. An additional two MPAs and two AGRs were omitted because we found that they were located outside of the study’s spatial scope of China’s UN-defined EEZ (Figs. 1 and 3), particularly the Paracel Islands (or Xisha Islands) where offshore military bases and exercises are common. This included the Xinan Zhongsha Bird Provincial Reserve, also known as the Xinan Zhongsha Archipelago Fishery Resource Reserve, which at 24,000 km^2^ would have been the largest MPA in the analysis. Full details on these MPAs and AGRs are available in a separate tab in the auxiliary dataset.

**Fig. 4.**
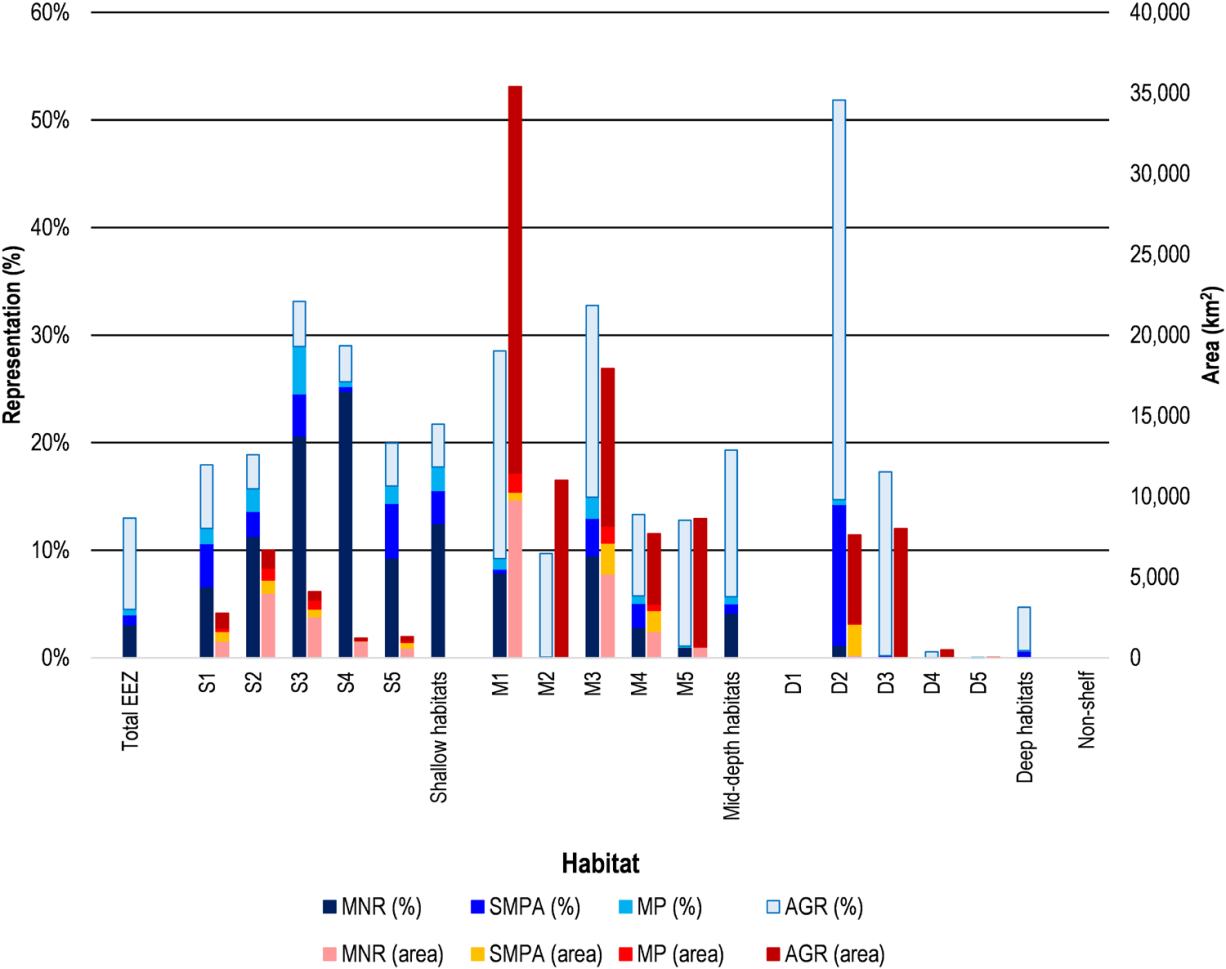
Representation of area-based conservation across classified habitats.

We measured “ecological representativeness” or representation as the percentage of a habitat’s total area (km^2^) that is located within protected areas. A lower percentage indicates a habitat with weaker representation, and a higher percentage indicates a more strongly represented one, with a greater proportion of its area contained within MPAs and AGRs. This evaluation method has been used in prior works assessing ecological representation of MPA networks (*26*, *31*, *32*). Some studies have used 10% as a threshold for sufficient representation, but highly important habitats may call for more (*26*). Results are presented for all conservation areas (MPAs and AGRs), MPAs only, and across the three main types of MPAs: marine nature reserves, special marine protected areas, and marine parks (Tables 2 and 3 and Fig. 4). Unless specified, “area protected” refers to both AGRs and MPAs combined.

**Table 2.** Descriptive statistics for habitat extents and habitat representation (area and % protected). Separated by MPAs and AGRs combined and MPAs alone.

**Habitat**	**Total habitat area**	**% of study area**	**Protection from** **MPA and AGR area** **(km^2^)**	**Protection from** **MPAs and AGRs** **(%)**	**Protection from** **MPA only area** **(km^2^)**	**Protection from** **MPA only (%)**
Total study area	869,427	100.00%	112,867	12.98%	39,143	4.50%
Shallow habitats	73,916	8.50%	16,061	21.73%	13,112	17.74%
S1	15,406	1.77%	2,764	17.94%	1,853	12.03%
S2	35,423	4.07%	6,685	18.87%	5,563	15.70%
S3	12,353	1.42%	4,093	33.13%	3,576	28.95%
S4	4,173	0.48%	1,210	29.00%	1,071	25.68%
S5	6,561	0.75%	1,309	19.95%	1,049	15.98%
Mid-depth habitats	417,799	48.05%	80,665	19.31%	23,755	5.69%
M1	124,099	14.27%	35,402	28.53%	11,455	9.23%
M2	113,666	13.07%	11,008	9.68%	79	0.07%
M3	54,740	6.30%	17,929	32.75%	8,170	14.92%
M4	57,784	6.65%	7,700	13.32%	3,322	5.75%
M5	67,511	7.76%	8,626	12.78%	729	1.08%
Deep habitats	343,986	39.56%	16,142	4.69%	2,277	0.66%
D1	57,658	6.63%	–	0.00%	–	0.00%
D2	14,700	1.69%	7,624	51.86%	2,160	14.69%
D3	46,364	5.33%	8,012	17.28%	93	0.20%
D4	87,586	10.07%	482	0.55%	–	0.00%
D5	137,677	15.84%	24	0.02%	24	0.02%
Beyond self	33,726	3.88%	–	0.00%	–	0.00%

**Table 3.** Descriptive statistics for habitat extents and habitat representation (area and % protected). Separated by different types of MPAs; marine nature reserves, special marine protected areas, and marine parks.

**Habitat**	**Marine nature** **reserves** **(area km^2^)**	**Marine nature** **reserves (%)**	**Special marine** **protected areas** **(area km^2^)**	**Special marine** **protected areas** **(%)**	**Marine parks** **(area km^2^)**	**Marine parks (%)**
Total study area	26,762	3.08%	7996	0.92%	4385	0.50%
Shallow habitats	9,214	12.47%	2277	3.08%	1621	2.19%
S1	1,019	6.61%	619	4.02%	215	1.40%
S2	4,003	11.30%	820	2.32%	740	2.09%
S3	2,549	20.64%	485	3.93%	542	4.39%
S4	1,034	24.77%	19	0.45%	19	0.45%
S5	610	9.29%	334	5.09%	105	1.60%
Mid-depth habitats	17,366	4.16%	3699	0.89%	2690	0.64%
M1	9,785	7.88%	482	0.39%	1188	0.96%
M2	79	0.07%	–	0.00%	–	0.00%
M3	5,196	9.49%	1915	3.50%	1059	1.93%
M4	1,638	2.83%	1302	2.25%	382	0.66%
M5	669	0.99%	-	0.00%	60	0.09%
Deep habitats	182	0.05%	2020	0.59%	74	0.02%
D1	–	0.00%	–	0.00%	–	0.00%
D2	170	1.16%	1927	13.11%	63	0.43%
D3	–	0.00%	93	0.20%	–	0.00%
D4	–	0.00%	–	0.00%	–	0.00%
D5	12	0.01%	–	0.00%	11	0.01%
Beyond shelf	–	0.00%	–	0.00%	–	0.00%

Nationwide, 4.50% of China’s marine and coastal habitats were protected by MPAs, and a total of 12.98% were protected when also including AGRs. MPAs comprised approximately one-third of all area-based marine protection, and AGRs conserved the remaining two-thirds. Of MPAs, 26,762 km^2^ or 68.37% were protected by no-take marine nature reserves.

Shallow habitats (<10 m) had 21.73% of their area protected, with 17.74% by MPAs and 3.99% by AGRs. Representation was consistently strong across all shallow habitats, but tropical and subtropical habitats tended to have higher representation, with habitats S3 and S4 more than 25% protected. No-take marine nature reserves protected 12.47% of shallow habitats.

Habitats in medium depth waters of 10 to 50 m comprised nearly half of all marine and coastal habitats in China by area, and 19.31% of their area were protected. In contrast to shallower habitats, protection of medium depth waters was mostly achieved through AGRs rather than MPAs. Habitats M1 and M3 were the most highly represented among these habitats, both exceeding 30% protection. These two habitats also had the highest chlorophyll concentrations, indicating that they were the most productive habitats in the medium depth zone.

Habitats in waters deeper than 50 m encompassed 40% of marine and coastal ecosystems in China. These habitats were largely underrepresented by MPAs and AGRs that protected only 4.69% of this zone combined, with less than 1% protected by MPAs alone. Of the area protected by MPAs, most were protected by multi-use special MPAs with only 170 km^2^ or 7.99% were located within no-take marine nature reserves. Protection of waters deeper than 50 m across habitats was the most inconsistent of any depth zone. As was the case for the medium depth zone, the most highly represented habitats—D2 and D3—were also the most productive habitats with the highest chlorophyll concentrations. Primarily located offshore from the mouth of the Yangtze and Qiantang rivers, 51.86% of habitat D2 was protected, making it the most highly represented habitat in the entire study. Much of this protection was in AGRs near important fishing grounds, such as the 13,500 km^2^ Lusi Fishing Grounds Fishery Conservation Zone (ID #22; see corresponding dataset). Habitat D3, primarily located south and east of D2, was also strongly represented with 17.28% of it being protected. Less than 1% of all other habitats in waters over 50 m depth were protected by MPAs or AGRs.

Habitats in the three primary depth zones were all located within the continental shelf, whereas 3.88% of China’s marine and coastal ecosystems extended beyond the continental shelf. These waters were mostly in tropical seas southeast of Hainan and close to Taiwan (Fig. 1). Visual analysis of the geomorphic database (*30*) against the shapefile for China’s UN-defined EEZ found that this region contained some critically important habitats for marine biodiversity including underwater canyons and seamounts. However, no protection was afforded to this region by MPAs or AGRs within China’s EEZ.

### Conservation objectives

We recorded the conservation objectives for each MPA and AGR (see the “Determining conservation objectives” section in the Supplementary Materials). While the MPAs provide equal protection to all biodiversity within, these conservation objectives reflected ecosystems and species of special focus or importance in the area. Multiple objectives were often attributed to a single site although were not stated or available for all locations. Of the 298 MPAs and AGRs that could be included in the analysis, 216 had more than one conservation objective and 16 had no recorded objectives.

Objectives were analyzed across two tiers of aggregated data because of inconsistent detail, syntax, and spelling of objectives across sites (Tables 4 and 5). Several sites had objectives that were not related to biodiversity or ecological representativeness (e.g., “ecotourism” or “temples”) that were not included among these aggregations (see the “Determining conservation objectives” section in the Supplementary Materials). The raw data are available in the supplementary dataset (see Data and materials availability for access). Aggregate 1 reflects general types of ecosystems (e.g., mangroves and coral reefs) or types of biota (e.g., fin fish, shellfish, and marine mammals), while aggregate 2 reflects common names of species or groups of species for a general audience (e.g., different species of horseshoe crabs are aggregated under one heading, “horseshoe crabs”). The tables reflect the most common objectives by frequency and area protected (the sum of areas of the MPAs and AGRs that included these objectives). Table 5 reflects the top 25 most common objectives for aggregate 2 for area and frequency.

Conservation of fin fish, shellfish, and birds was the most common objectives for aggregate 1 results by frequency and area protected across MPAs and AGRs combined. This was especially true for AGRs, which of 51 AGRs, 44 included shellfish and 30 included fin fish among their objectives (Table 4). Few AGRs included objectives beyond these two categories.

Results for aggregate 2 demonstrate that commercially important species were the most common objectives for protection, again primarily within AGRs. Fin fish such as croaker (*n* = 8, 36,606 km^2^), cutlassfish (*n* = 4, 33,982 km^2^), and butterfish (*n* = 13, 500 km^2^) were the most common by area under protection (Table 4A), and shellfish such as clams (*n* = 15, 565 km^2^), abalone (*n* = 10, 50 km^2^), sea urchins (*n* = 12, 212 km^2^), and lobster (*n* = 9, 985 km^2^) were the most common by frequency, although rare or endangered species like sturgeon were occasionally objectives for AGRs as well (*n* = 3, 176 km^2^). However, MPAs more commonly included rarer or endangered species among their objectives such as horseshoe crabs (*n* = 10, 227 km^2^) and fin fish such as sturgeon (*n* = 3, 787 km^2^). Some MPAs also included forage fish among their objectives (*n* = 10, 1412 km^2^)—including lancelets, anchovies, and mackerel (but not Spanish mackerel)—that provide important supporting roles for marine ecosystems and biodiversity (*33*).

**Table 4.** Aggregate 1 conservation objectives in order of total area protected (km^2^, highest to lowest) and frequency (highest to lowest), including breakdown by AGRs and MPAs. marine nature reserves, MNRs; special marine protected areas, SMPAs; and marine parks, MPs.

**A. Aggregate 1** **category**	**Total area**	**AGR area**	**MNR area**	**SMPA area**	**MP area**
Fin fish	54,571	48,996	2,319	2486	770
Shellfish	40,154	36,254	2,355	1192	353
Birds	10,415	8	10,066	243	98
Marine mammals	10,253	20	10,220	–	14
Wetlands	9,450	–	7,764	660	1026
Marine ecosystem	4,009	–	997	1845	1168
Coastal ecosystem	1,775	–	115	564	1095
Coral reef	1,016	–	382	549	85
Mangroves	846	–	644	85	117
Plants and algae	601	45	422	41	92
Other invertebrates	227	207	20	–	–
**B. Aggregate 1** **category**	**Total freq.**	**AGR freq.**	**MNR freq.**	**SMPA freq.**	**MP freq.**
Shellfish	84	44	25	14	1
Fin fish	60	30	17	8	5
Birds	52	1	43	3	5
Marine ecosystem	42	–	22	8	12
Wetlands	40	–	22	5	13
Mangroves	38	–	31	3	4
Coastal ecosystem	20	–	4	3	13
Marine mammals	19	1	16	–	2
Plants and algae	14	4	6	2	2
Coral reef	10	–	7	1	2
Other invertebrates	9	7	2	–	–

**Table 5.** Aggregate 2 conservation objectives in order of area protected (km^2^) and frequency, highest to lowest for top 25 results, including breakdown by AGRs and MPAs.

**Rank**	**Aggregate 2** **category**	**Total frequency**	**AGR frequency**	**MNR frequency**	**SMPA frequency**	**MP frequency**
1	Croaker	39,285	36,607	492	1,983	203
2	Cutlassfish	33,982	33,982	–	–	–
3	Butterfish	13,500	13,500	–	–	–
4	Shrimp and prawns	11,802	11,802	–	–	–
5	Bream	11,606	11,606	–	–	–
6	Carp	11,488	11,470	–	18	–
7	Seals	8,454	–	8454	–	–
8	Forage fish	1,821	410	917	36	459
9	White dolphin	1,370	–	1368	–	2
10	Crabs	1,339	216	1055	68	–
11	Oysters	1,315	78	1171	49	17
12	Lobsters	1,047	985	49	13	–
13	Clams	1,005	565	157	282	–
14	Sturgeon	964	176	787	–	–
15	Cranes	815	–	815	–	–
16	Gulls	800	–	800	–	–
17	Abalone	776	250	55	13	459
18	Sea urchins	718	212	22	–	484
19	Flatfish	710	399	28	215	69
20	Egrets	578	–	578	–	–
21	Dugongs	374	–	363	–	11
22	Snails	348	332	16	–	–
23	Eels	245	245	–	–	–
24	Horseshoe crabs	227	–	66	90	71
25	Mullet	220	220	–	–	–
**Rank**	**Aggregate 2** **category**	**Total frequency**	**AGR frequency**	**MNR frequency**	**SMPA frequency**	**MP frequency**
1	Clams	19	15	2	2	0
2	Abalone	17	10	5	1	1
3	Sea urchins	17	12	3	0	2
4	Lobsters	15	9	5	1	0
5	Flatfish	14	9	1	3	1
6	Croaker	13	8	2	2	1
7	Forage fish	12	2	7	2	1
8	White dolphin	12	0	11	0	1
9	Horseshoe crabs	10	0	7	1	2
10	Oysters	10	4	3	2	1
11	Snails	9	8	1	0	0
12	Crabs	8	5	2	1	0
13	Egrets	7	0	7	0	0
14	Shrimp and prawns	7	7	0	0	0
15	Sea cucumber	6	4	2	0	0
16	Sturgeon	6	3	3	0	0
17	Cutlassfish	4	4	0	0	0
18	Eels	4	4	0	0	0
19	Mullet	4	4	0	0	0
20	Pen shell	4	3	1	0	0
21	Scallops	4	4	0	0	0
22	Spanish mackerel	4	4	0	0	0
23	Bass	3	3	0	0	0
24	Bream	3	3	0	0	0
25	Carp	3	2	0	1	0

MPAs also included a wider diversity of charismatic megafauna among conservation objectives including birds (*n* = 51), such as egrets (*n* = 7, 578 km^2^), cranes (815 km^2^), and gulls (800 km^2^). Marine mammals (*n* = 18) were also common conservation objectives for MPAs, especially the Chinese white dolphin (*n* = 12, 1370 km^2^), seals (8454 km^2^), and dugongs (374 km^2^). Sea turtles (*n* = 2) and a type of finless porpoise (*n* = 1), likely a marine subspecies of *Neophocaena phocaenoides* (*35*), were also objectives but were not captured among the top 25 in Table 5. In addition to specific species, MPAs also commonly included important marine ecosystems among their stated conservation objectives, including wetlands (*n* = 40), mangroves (*n* = 38), and coral reefs (*n* = 10), and undefined ecosystems reflected as “marine ecosystems” (*n* = 42) and “coastal ecosystems” (*n* = 20).

## DISCUSSION

### Ecological representativeness

#### Future directions for marine protection in China

Our analysis found that China’s MPAs and AGRs have provided relatively robust protection across shallower habitats near and along the immediate coast. These shallower habitats were mostly protected by fully no-take MPAs that prioritize protection of a wide diversity of marine life across numerous ecosystems and that include vulnerable and endangered species such as the Chinese white dolphin and finless porpoise, respectively. Other frequently protected biota included culturally valuable animals such as birds and horseshoe crabs and groups of species that play key supporting roles for their wider ecosystems like forage fish (*33*). Shallow-habitat MPAs also protected important coastal ecosystems that have been under great historical pressure from coastal development including mangroves, coastal wetlands, and coral reefs (*35*). Other degraded coastal ecosystems, such as seagrass beds, were less frequently included among conservation objectives, but that does not mean that they are not protected.

A smaller proportion of habitats occurring further from shore in waters 10 to 50 m and >50 m deep were protected than relatively shallow habitats. Protection levels also became more inconsistent across habitats as depth increased. Some habitats had very high protected area coverage, especially more productive habitats with high chlorophyll concentrations. Habitats with lower levels of productivity were afforded little protection. Protection also became increasingly dominated by AGRs over MPAs as depth increased. Of the limited area protected by MPAs, this was almost all within special marine protected areas and marine parks that are multi-use and less restrictive than fully no-take marine nature reserves.

Our findings demonstrate that China has made great efforts to protect some types of marine habitats in the country, but there are substantial gaps in conservation of others. As China looks to expand and strengthen its domestic conservation efforts (*18*, *19*), it would be prudent to increase protection of habitats that are currently poorly protected.

Underrepresented habitats include tropical pelagic seas and important ecosystems beyond the continental shelf such as underwater canyons and seamounts. Habitats that have received substantial protection from AGRs might be considered for upgrading to MPAs in the future. Recent publications have indicated that seas west and southwest of Taiwan and offshore of Shandong Province currently receive little to no protection from MPAs or AGRs, yet they are among the most important parts of the global ocean to protect. These patterns contrast from other MPA networks of large countries in the Pacific and Indian Ocean regions, such as Australia where offshore tropical waters are the most highly represented, demonstrating how patterns and gaps in ecological representativity can vary across countries (*32*).

It can be logistically challenging and financially costly to protect ecosystems that are far from shore (*36*). As target areas for China’s industrial fishing activity (*37*), it could also be politically challenging. However, protecting these important regions could be feasible with, for example, substantial support from China’s national government.

Additional protection for relatively well-represented habitats should also be considered. Global targets to protect the ocean may soon be substantially increased above the currently agreed 10%, and thus, additional protection of highly important ecosystems such as mangrove forests, seagrass beds, coastal wetlands, and coral reefs may be required to meet future global commitments. While many MPAs already prioritize these ecosystems as conservation objectives (see the “Conservation objectives” section in Results), increasing the area protected through expansion and fortification of the MPA network would help to ameliorate the substantial loss of natural shorelines because of coastal development. For example, 77 to 87% of mangroves have been lost in China since 1950 (*35*).

### Progress toward achieving global targets

This study is one of the first to analyze China’s progress toward meeting globally agreed targets for protecting the ocean, and the first to directly consider AGRs as part of China’s area-based conservation network. We found that when only MPAs are considered toward global targets, only 4.50% of marine and coastal habitats are protected, which falls far short of the Aichi target of 10% protection by 2020. Other recent publications have similarly estimated that about 5% of marine environments in China are protected by MPAs (*18*–*20*), although some differ in spatial scope from this study by incorporating a larger area of claimed territorial seas than the UN-defined EEZ. If AGRs are included in the statistics, then China may meet or even modestly exceed the current target, depending on the extent to which AGRs qualify as OECMs.

### Study limitations

There were limited data available on China’s marine environments and site-specific information for MPAs and AGRs. For environmental data, from which the series of habitats were classified, a lack of publicly available spatial information on China’s marine ecosystems required us to use remote sensing and geomorphic datasets for most of the analysis. Some prior studies assessing MPA ecological representativeness have used global spatial data on key ecosystems like mangroves, coral reefs, and seagrass beds (*26*). These were not included because, as a comprehensive nationwide study, we focused on variables and databases that were equally distributed and available across the entire study area. These ecosystems are also relatively small and require a high degree of resolution to adequately capture. Therefore, it may not be appropriate to conduct an overlap analysis with the lack of spatial data available for China’s MPAs that required estimating their boundaries and many of their locations. These more specific ecosystems could be better incorporated with improved spatial data of China’s MPAs. However, until those data become available, we were able to provide meaningful insight on the prevalence of these ecosystems within China’s MPAs via the analysis of conservation objectives.

Prior studies have also used sediment type to inform ecological representativeness of MPA networks (*32*, *38*). We attempted to include sediment type as well, but data for China were only available across small independent studies, and there were no data sources available—Chinese or international—that encompassed the entire EEZ. Despite these data limitations, the complexity and quality of the information and habitat classification in our study is comparable to several other studies analyzing ecological representativeness of marine protection in other countries (*31*, *32*, *38*, *39*).

We were also limited by the lack of available site-specific information for some MPAs and AGRs in China. An extensive review of several different sources with varying degrees of detail and relevance was conducted from which a master list of 273 MPAs and 53 AGRs in the country was developed and upon which this study was based (see Materials and Methods). Detailed information including area, location, and other variables was collected for all 53 AGRs and 249 of the MPAs available. The 24 MPAs for which area and location were not available were known to be municipal or county/locally managed areas. The municipal and local/county managed areas that were included in the analysis (*n* = 90) averaged 55.6 km^2^ in area with a median of 10.32 km^2^ compared to a sample wide average (all MPAs at all management levels including municipal and local/county) of 253.59 km^2^ and median of 25.4 km^2^. Thus, the MPAs that were omitted because of data limitations were of a subgroup (municipal and county/local managed areas) that is generally much smaller than national or provincial MPAs, and their exclusion is unlikely to have had a significant impact on the study’s results and conclusions.

The lack of spatial data on MPAs also limited us from evaluating the degree of potential overlap across MPAs and AGRs, which has occurred for some sites before the 2018 Institutional Reform. These cases are rare and historical; when an overlap was detected, it was generally followed by amended legislation to clarify the boundaries. Therefore, any potential overlap of MPAs and AGRs is not likely to significantly affect results.

Furthermore, we focused our analysis on MPAs within the study’s spatial scope as outlined by the UN-defined EEZ. China-based resources indicated that there are MPAs under management outside of these boundaries in the South China Sea. We did include these MPAs in our maps (Figs. 1 and 2) and publicly available website, and details are also available within a separate tab in data S1 (see “Data and materials availability”). However, these MPAs were not directly incorporated into our analysis of ecological representation and conservation objectives, which required defined spatial scopes. Some, such as the 24,000 km^2^ Xinan Zhongsha Bird Provincial Reserve, also known as the Xinan Zhongsha Archipelago Fishery Resource Reserve (MPA ID #149), were substantial and could provide protection for some of the deep water tropical habitats where protection was found to be lacking. However, this would also require greatly expanding the assumed spatial area of China’s seas and marine ecosystems, which would mitigate the impact of adding these MPAs to the analysis and would not likely change the overall outcome and main takeaways from the research.

Last, this research provides important recommendations for improving marine conservation through the analysis of ecological representativeness and distribution of different types of area-based conservation. However, it does not assess the effectiveness of site-level management that is crucial to the success of MPA networks and is frequently insufficient on a global level (*40*). Other global studies have analyzed factors known to indicate the potential effectiveness of MPAs, such as no-take status, age, and others that found few MPAs in China meet these characteristics (*18*, *41*). However, research on the capacity and effectiveness of management practices for MPAs and potential OECMs in China remains largely understudied and is an important area for future research.

Our analysis of area-based marine protection in China outlined strengths and priorities among the existing network of MPAs and AGRs while also identifying gaps and potential pathways for improvement. In what may be contrary to many expectations, China extends substantial protection to some types of marine environments. This is especially true for ecosystems near and along the immediate coast such as mangroves and important habitats for commercially important species of fin fish and shellfish, as well as culturally important charismatic megafauna including birds and marine mammals. Most of the network of MPAs is also composed of fully protected MPAs nationwide where fishing is not allowed.

However, we found large gaps in protection—especially from fully no-take MPAs—for habitats in deeper waters further from shore, especially tropical pelagic ecosystems. Benefits of protection from climate mitigation in these offshore habitats—in addition to carbon sequestered in “blue carbon” ecosystems including mangroves, wetlands, and seagrasses—mean that marine protection in China could play an important role in meeting the recent pledge to achieve carbon neutrality by 2060 (*35*, *42*). Protecting these deeper waters will also be increasingly important to protect against deep-sea mining that presents a major future threat to the ocean with rising demand for metals (*43*, *44*). Furthermore, because of historic precedence for MPAs in shallower coastal waters, expanding protection may require new policies and management frameworks for adapting MPAs to these new environments further from shore.

We also found that China may have met or even exceeded the goals to protect 10% of marine and coastal environments by the year 2020 within its territorial seas if AGRs are counted as OECMs. There is still substantial room for improvement, and expansion of China’s area-based conservation network may be required if internationally agreed targets substantially increase as a result of international negotiations later this or next year. Our analysis identifies which habitats are most in need of additional protection in order to achieve ecologically representative conservation. MPAs and AGRs in China can also play an important role for connectivity of marine protection networks in the western Pacific Ocean, which should be factored into decision making for new and expanded MPAs.

This extensive collaboration between the United States and China–based researchers has painstakingly compiled from many disparate sources the first comprehensive publicly available database to date on marine protection in China. It includes names of all 273 known MPAs and detailed information on size, location, conservation objectives, and other attributes for 249 MPAs and 53 AGRs (see auxiliary dataset). By making this database available, including via an online web platform for viewing and exploring the map (see “Data and materials availability”), we hope to advance the international community’s understanding of China’s domestic marine conservation efforts and to stimulate further research. The database could also be a valuable resource for the Chinese government and its ambitions for improving the network following the 2018 institutional reform that brought all MPAs under one government agency (*18*, *19*). In the meantime, our analysis provides an initial roadmap to improve the ecological representativeness of the area-based conservation network.

## MATERIALS AND METHODS

### MPA data collection

#### Compiling a list of 273 MPAs and 53 AGRs

It was necessary to compile our own list of MPAs and AGRs from multiple sources because there was no publicly available resource that included all of China’s conservation areas. We initially developed two separate lists simultaneously using different methods that were then cross-compared to develop a refined and vetted list of MPAs and AGRs.

The first list was compiled remotely by reviewing an array of Chinese language sources, including:

1) *Chinese marine protected areas* by Zeng (*45*)

2) Government protected area list by the Chinese Ministry of the Environment (*46*)

3) Government aquatic germplasm reserve list (*47*)

4) *Oceanol* (www.oceanol.com)—A Chinese website for marine conservation news and reports. (*Oceanol* was taken offline between May and June 2020).

These sources were reviewed between November 2019 and July 2020. We separated marine from terrestrial PAs by removing all PAs from landlocked provinces and visualizing the locations on Google Maps and ArcGIS. We also cross-referenced all sources to remove duplicate entries.

The second list of MPAs and AGRs was developed in parallel through phone and email consultations with representatives at the relevant governmental institutions for MPAs and AGRs, including management organizations, local governments, and other government agencies involved in MPA and AGR management. The name, type of MPA/AGR, and governance for each site were determined by cross-verifying information across these sources in addition to internal government documents and news reports describing their work.

Each MPA and AGR from the two lists was then individually cross-compared to identify matching or corresponding sites. There were initially many discrepancies between the two lists, mostly due to MPAs that had been given two or more names, which was a common occurrence before the 2018 Institutional Reform. Discrepancies were resolved with further consultations among team members and government agency personnel until the two lists matched one another. This method developed a refined, robustly vetted list of 273 MPAs and 53 AGRs with no duplicates (see data S1). Names from both the remote and expert consultation process were kept and made available in data S1 as Chinese/English name 1 and Chinese/English name 2, respectively.

Analyzing ecological representativeness required additional data for area, location (coordinates), spatial extent, and conservation objectives of each MPA and AGR. The first four resources used to develop the list of names were insufficient and, in some cases, outdated for these variables. Additional information was gathered by conducting Google and Google Scholar searches for each site. New databases or sources of information were also consulted including the web platforms Baidu, Osgeo, and China Mangrove. We also reviewed news articles about the MPAs and AGRs. The Supplementary Materials provide more context on these sources of information, and specific webpages are cited for the relevant data points in the auxiliary file.

The spatial extent of each MPA and AGR was needed to identify which habitats were protected within their boundaries. A lack of maps and other detailed spatial information for many sites required us to estimate their spatial extent (see the “Study limitations” section in Discussion). We used available coordinates to mark the center of each site, around which a radius was drawn based on the site’s known area (km^2^). Coordinates were available for MPAs and AGRs that comprised most (77.4%) of the area protected, with coordinates for the remaining sites determined by one of four alternative methods (see the “Determining coordinates for each MPA and AGR” section in the Supplementary Materials). Methods for determining conservation objectives from source material and organizing and aggregating them into aggregate groups are discussed in the supplementary material (see the “Determining conservation objectives” section in the Supplementary Materials).

### Habitat clustering and identification

Marine habitats in China were defined through a series of *k*-means cluster analyses that segmented marine areas into representative groups based on sea surface conditions across three different depth zones. The two-step clustering method was adapted from a global study that identified representative groups of MPAs (*9*). Other studies of MPA ecological representativeness have similarly combined principal component analyses with *k*-means clustering to define habitats (*32*). The method identified 16 distinct habitats based on average annual sea surface temperature (°C), chlorophyll-α concentration (mg/m^3^) as a proxy for primary productivity, and depth (m). Further specifics of the methods and analysis for identifying habitats, including variable selection and data collection, are discussed in the Supplementary Materials (see the “Clustering approach and habitat identification” section).

### Analyzing ecological representativeness

Representation for each habitat was defined as the percentage of the habitat protected by MPAs and AGRs. Most of the area within MPAs and AGRs (~80%) could automatically be assigned to a habitat based on where they overlapped. The remainder (~20%) of area protected required additional steps for habitat assignment (see the “Assigning habitats” section in the Supplementary Materials)

After all of the protected area within MPAs and AGRs had been assigned to one of the 16 habitats, we calculated representativeness via the following equations

$$\text{Representation under MPAs}=100\times \frac{\text{Total area of MPAs assigned to habitat}\;({\text{km}}^{2})}{\text{Total habitat area}}$$ (1)

$$\;\text{Representation under AGRs}=100\times \frac{\text{Total area of AGRs assigned to habitat}\;({\text{km}}^{2})}{\text{Total habitat area}}$$ (2)

The area for each habitat was calculated using the following equation

$$\text{Habitat Area}=100\times \frac{\#\text{of bathymetry data points within the habitat}}{\text{Total}\#\;\text{of bathymetry data points in}\;\text{EEZ}}$$ (3)

“Study area” was calculated from the digitized EEZ polygon-shape file (from Esri and the Flanders Marine Institute) in square kilometers (see the “Interpolation” section in the Supplementary Materials). We used ratios of bathymetric points to measure the habitat proportions because they were evenly distributed throughout the study area (see the “Interpolation” section in the Supplementary Materials).

## Supplementary Materials

### This PDF file includes:

Supplementary Text Figs. S1 and S2 Tables S1 and S2 References
