# Supplementary material for: China’s little-known efforts to protect its marine ecosystems safeguard some habitats but omit others
Source: Sci Adv. 2021 Nov 12;7(46):eabj1569. doi: 10.1126/sciadv.abj1569 (PMC8589307; doi:10.1126/sciadv.abj1569)
Supplement: Supplementary file 1 — Supplementary Text Figs. S1 and S2 Tables S1 and S2 References [file sciadv.abj1569_sm.pdf]

Science Advances
AAAS

Supplementary Materials for

China's little-known efforts to protect its marine ecosystems safeguard some habitats but omit others

John J. Bohorquez, Guifang Xue, Timothy Frankstone, Maria M. Grima, Karine Kleinhaus, Yiyi Zhao, Ellen K. Pikitch*

*Corresponding author. Email: ellen.pikitch@stonybrook.edu

Published 12 November 2021, *Sci. Adv.* **7**, eabj1569 (2021)
DOI: 10.1126/sciadv.abj1569

This PDF file includes:

Supplementary Text
Figs. S1 and S2
Tables S1 and S2
References

Supplementary Text

1. Methods

1.1. Data collection for MPAs and AGRs

1.1.1. Resources

Data for year of establishment, area, location, and conservation objectives of each MPA and AGR were collected from a wide array of Chinese language resources. Some sources reported conflicting information about the MPAs and AGRs, so the resources were prioritized to decide which information to include into our dataset according to the following ranking order:

1. Government PA and AGR lists *(46, 47)*: Official government sources were always considered to be the most reliable.
2. Oceanol: The news outlet included articles and profiles of specific MPAs, including detailed descriptions, coordinates, and area of the sites. This source was regularly updated and included detailed information on SMPAs and MPAs not included in the government lists.
3. National Aquatic Resources Collection System (http://zyyh.cnfm.com.cn/): While this website had information on relatively few AGRs, it provided an organized system for recording the coordinates and area of these sites.
4. Baidu (baike.baidu.com): This platform, a China-based online encyclopedia, provided highly detailed information on conservation objectives and location for many MPAs, but was not government officiated.
5. Other websites (e.g., China Mangrove, Osgeo, Chinese news sites): While these other websites often contained only very general information on MPAs, they contained missing information for some sites.
6. Zeng 2013 (45): Although one of the first sources used in compiling the list of MPAs, this source dated from 2013 and was compiled independently rather than directly from government sources. Many MPAs had been expanded since its publication.

When a conflict between two sources arose, the data from the higher-priority source were used in the database. All sources of information were recorded for each variable of each MPA and AGR and are listed in the corresponding dataset.

1.1.2. Determining conservation objectives

Conservation objectives were identified based on priorities explicitly stated for MPAs, either in a dedicated “conservation objective” entry in the source material or in the name of the area (e.g., “Wuli Nanshan Mangroves”). The conservation objectives were recorded for each MPA and AGR at the highest level of detail provided in the source material. The raw data is available in a separate tab in the auxiliary dataset.

There were varying degrees of detail and syntax used to communicate objectives. Some listed very specific species and even scientific names in some cases (e.g. *Atrina pectinata)*, others included more general common names (e.g. horseshoe crabs) or groups of species (e.g. marine mammals). Others referred to

broader ecosystems (e.g. mangroves), terms of use (e.g., ecotourism), and geological or archaeological features. Terms that were considered practically interchangeable were consolidated, such as ‘mangroves’, ‘mangrove habitat’, and ‘mangrove environment’ which were all grouped under the term ‘mangrove’. Terms like ‘mammals’, ‘marine mammals’, and ‘Dugongs’ were kept separate, as were different species or subspecies like ‘Southern Horseshoe Crabs’ versus ‘Chinese Horseshoe Crabs’. This process yielded a total of 142 unique conservation objectives across the MPAs and AGRs in the dataset, which were integrated into a matrix marking the presence ‘TRUE’ or absence ‘FALSE’ of each objective for each site. Objectives only indicate biodiversity and natural features that are of special importance for management and ‘absence’ of a species or feature from a given MPA or AGR does not mean it is not present nor protected by the site.

We then developed two tiers of aggregation, Aggregate 1 and Aggregate 2, to more succinctly organize, analyze, and present the 142 objective terms in a digestible manner (Tables 4 and 5 in the main body). The first, ‘Aggregate 1’, was used to represent the general types of environments (e.g., ‘mangroves,’ ‘coral reefs’) or types of marine organisms (e.g., ‘fin fish,’ ‘marine mammals’) that were of special importance or focus of the site. 11 unique terms were organized into Aggregate 1. Terms that were not specific to marine environments or biodiversity, such as ‘Ecotourism’, ‘Temples’, ‘Beaches’ and others were not included in the aggregation.

The second, ‘Aggregate 2’, was more detailed and identified common names for specific species (e.g., white dolphin), or groups of species (e.g., all species of clams, horseshoe crabs, and groupers were grouped under terms ‘clams’, ‘horseshoe crabs’, and ‘grouper’ respectively). Species were grouped in ways that would be communicable to a wide audience and did not follow a standard taxonomic approach. 62 unique terms were defined under Aggregate 2.

1.1.3. Determining coordinates for each MPA and AGR

We determined a single coordinate for each conservation area, which served as a centroid from which a radius was drawn to represent the spatial extent of the site. All spatial analyses were conducted in ArcGIS Desktop 10.7.1 software by ESRI *(49)* using China Geodetic Coordinate System 2000, China’s officially recognized system *(50)*. Using the “Buffer tool” from the “Proximity toolset” of the “Analysis toolbox,” each conservation area centroid was assigned a circular buffer according to its radius as derived from its known area.

Coordinates for each site were determined based on one of the following 5 methods, in ranked in order of priority:

(1) GIVEN: Centroids or coordinates provided. This method was used for 175 of 298 conservation areas included in the analysis, which collectively comprised 77.4% of the total protected area within the study’s spatial scope. A handful of sites provided a single coordinate, and those coordinates were applied as the centroids. However, most sources provided a range of coordinates that were used to calculate centroids by taking the means of the longitudinal and latitudinal extremes. Any coordinates provided in degrees, minutes and seconds were converted to decimal degrees before calculating centroids.

(2) NEAR GIVEN: Exact coordinates were not provided, but coordinates were found for a landmark within or near the conservation area’s boundaries. For example, coordinates provided for the Lusi Fishing Grounds were used to inform coordinates for the Lusi Fishing Grounds Fishery Conservation Zone (ID #22). This method produced centroids for 2 sites, comprising 12.0% of the total protected area.

(3) MAP: Coordinates were visually estimated using maps of the site that provided coordinates on the x and y scales. 2 MPA centroids, 0.6% of the total protected area, were processed by this method.

(4) GE (Google Earth): Searchable locations or landmarks were indicated by the name of the conservation area or a distinguishing feature nearby. The Google Earth search terms used for each of these locations are provided in the dataset. Centroids for 21 conservation areas, 1.0% of the total area, were determined using this method. Locations for areas outside of the spatial scope of the analysis (n = 4) were identified using this method. While these sites were not retained for final analysis, they are still made available with the supplementary dataset.

(5) GIS: Through the ArcMap data portal, a polygon shapefile was found for "China County City District Boundaries 2016" that contained locations and associated location codes (Table S2). Since location names (e.g. city, county, etc.) could be derived for 100% of conservation areas in the database, the retrieved locations were compared with the locations listed in the shapefile's table. Each prospective location polygon was visually assessed to ensure that it was on or near the coast, and in the correct province and sea. Furthermore, this method was never applied for cases where the conservation objective of a given MPA or AGR indicated that it was located offshore. For the conservation areas that passed the tests, a field was added to the dataset containing the shapefile's corresponding location code. A join was then executed between the set and the polygon shapefile based upon this location code field. Once joined, the centroid of each polygon was calculated and snapped to the nearest coastline. This method provided coordinates for 98 conservation areas, comprising 8.9% of the total protected area in the dataset.

Within the 249 MPAs and 53 AGRs that were included (247 MPAs and 51 AGRs within the study's focal area), there were some data limitations concerning the expanse and locations of some sites. For example, many MPAs have terrestrial areas included in their total size, but only one database (Oceanol) consistently distinguished marine and terrestrial area for MPAs that included dry land. Of the 67 MPAs listed on Oceanol, 27 reported having a terrestrial component. The sum of the total terrestrial area (km^2) within those 27 MPAs comprised only 4.9% of total cumulative area. Therefore, marine area (km^2) would be overestimated by ~5% had we assumed all 67 of these areas were 100% marine. As a result, considering Oceanol as a representative sample, it is unlikely that assuming the same ratio of land and sea for the rest of the MPAs and AGRs would lead to a significant overestimate of protected area in the analysis. Moreover, it is unlikely that MPAs or AGRs situated far from the coastline would contain any terrestrial component. We therefore proceeded with the assumption that 100% of reported area for all sites in the study as marine.

1.1.4. Assigning habitats

Polygons of each habitat were created using the cluster outputs from the PCA and k-means analyses (supplementary material section 3.2.) using the "Aggregate Points" tool from the "Generalization toolset" of the "Cartography toolbox" in ArcGIS. An aggregation distance was set for 0.015646 decimal degrees to eliminate the possibility of standalone points yet allow diagonals to be drawn. Once the polygons for the 16 classified habitats were developed (Figure S1), we measured how they were distributed within the estimated boundaries for the MPAs and AGRs.

Habitats within MPAs and AGRs were identified using a two-step process. First, habitats within each site were evaluated using the "Tabulate Intersection" tool in the "Statistics toolset" of the "Analysis toolbox," by calculating area within each MPA and AGR that intersected with an individual classified habitat. This

approach assigned 79.8% of the total area within MPAs and AGRs in the study to a specific habitat. It was not able to initially assign 100% of all MPAs and AGRs to a habitat because of limited refinement of available benthic and satellite data, especially along the coastline where data gaps were most prevalent with intrusion of dry land. Some MPAs and AGRs did not overlap any assigned habitats due to such limitations. Furthermore, the radian approach to estimating the extent of each MPA and AGR meant that the estimated range for many areas near the coast overlapped terrestrial zones.

The second step for assigning habitats to the remaining 20.2% of area within MPAs and AGRs incorporated one of two methods depending on whether (1) some, but not all, of the MPA or AGR was already assigned to one or more classified habitats in the first step, or (2) none of the MPA or AGR was initially assigned to one or more habitats in the first step.

For conservation areas falling under the first scenario where part but not all the area was assigned, we extrapolated the existing habitats proportionately to fill in the rest of that area. For example, if the first step only assigned 25% of an MPA to a specific habitat, 10% L1 and 15% to M1, then the rest of the unassigned area would be allocated to each of those habitats proportionately, such that a total of 40% was ultimately allocated to L1 and 60% to M1. This process of multiplying proportionate habitat allocations assigned habitats to 16.2% of area protected.

The remaining 4.0% of area in the dataset was contained within MPAs and AGRs that did not have any assigned habitats within their boundaries. For these cases, the MPAs and AGRs were manually assigned to the habitat that was closest to the centroid of the respective site. Only 1 habitat was assigned to each area through this process.

1.2. Clustering approach and habitat identification

1.2.1. Data collection and consolidation

Four variables were considered for sea surface conditions that prior studies have incorporated for identifying habitats via remote sensing (Table S1): Sea Surface Temperature (SST, ^{o}C), Sea Surface Temperature Range, Sea Surface Salinity (SSS, psu), and chlorophyll-α concentration (chl.-α, mg/m^3) *(51, 52)*. SST, SSS, and chl.-α were directly sourced from NASA's Earth Observations (NEO) database *(53)*, a publicly available record of global satellite remote sensing data.

The global dataset was trimmed down to boundaries between 15 – 40° North, and 107 – 127° East to fully capture the extent of the assumed study area. Data for each variable was collected via monthly averages from 2010-2019 at 0.1-degree resolution for SST and chl.-α, and a 0.5-degree resolution for SSS. Seasonal and annual averages were calculated from these monthly averages for each observation point in the 10-year dataset. Seasonal averages were included to open the analysis to the possibility that spatial differences may be more pronounced at different times of the year. Seasons were defined in 3-month periods, such that December through February was designated as winter, March through May as spring, June through August as summer, and September through November as autumn. SST range was calculated on an annual basis as the difference between the highest and lowest monthly average for a given year for each point.

Cloud cover, seasonal ice (which is known to occur in parts of the Bohai Sea *(54)*), and other environmental conditions can sometimes prevent the collection of remote sensing data at specific locations. We were not able to collect monthly average measurements for all 120 months in the 10-year dataset for the majority of spatial data points, but more than 90% of data points had measurable data for at least 90 months. All available data within the study scope were used in the interpolation step, even locations where data availability was intermittent. However, to prioritize the locations that were more consistently measured, the interpolation included a weight for frequency of reporting.

1.2.2. Interpolation

A polygon shapefile of China's exclusive economic zone (the "study area") as taken from the Flanders Marine Institute (48), and the different depth zones and non-shelf zone within, were created before beginning the interpolation process. The relevant boundaries of the "World Exclusive Economic Zone Boundaries" feature layer (Table S2) were digitized to form the seaward bound of the polygon. In the near-negligible areas where that EEZ did not meet the coast, the "Exclusive Economic Zones of Flanders Marine Institute _2018_ v_10" was used (Table S2). Excess area was then clipped to the "China Country Boundary 2018" (Table S2) to form the coastal bound of the polygon for the study area. To discriminate the shelf and non-shelf area portion, the areas beyond the continental shelf of the "World Seafloor Geomorphology" (Table S2) were digitized at a minimum scale of 1:63,360 and clipped the resulting polygon shapefile to the study area. A polygon of the continental shelf was then created using the difference between the non-shelf and study area polygons.

The "Seafloor Bathymetry (meters) – Transparent" raster file (Table S2, *(55)*) was imported to obtain depth points. The "Raster to Point" tool from the "Conversion toolbox" was used to convert the data into point form, which was then clipped to the shelf. Each cell of the raster produced a datapoint located in the center of the cell. Because of their even distribution, the data points were later used to estimate the proportion of habitats within the study area (see section 5.3.2).

Point shapefiles were generated from each of the 16 remote sensing tables. After clipping to the EEZ, the "Inverse Distance Weighting" (IDW) tool from the "Geostatistical toolbox" was used to interpolate each variable, using the frequency of reporting to weight the results. IDW uses known spatial values to predict unknown spatial values. It assumes that locations closer to the measurement points will have more similar characteristics than those further away. This method was chosen since it is applicable to large datasets and is bound by pre-existing minima and maxima *(56)*. Since the resolution of the SSS remote sensing data was lower than the rest, its interpolated rasters did not cover the Bohai Sea nor northern areas of the Yellow Sea. For this reason, salinity was then removed from further analysis.

For each of the remaining 11 IDW rasters, the "Extract Multi Values to Points" tool from the "Spatial Analyst toolbox" was applied using the bathymetry shapefile as the input point feature. This created a new point shapefile comprised of the IDW raster values that existed at a given bathymetric datapoint. These files were then downloaded using the "Table to Excel" tool for use in the PCA and k-means analyses.

Following interpolation 1,268,528 datapoints remained upon which k-means analyses were performed separately as follows: Shallow n = 112,199, Medium n = 634,186, Deep n = 522,143.

1.2.3. PCA and K-means analyses

PCA and k-means analyses were performed in R version 4.0.0 "Arbor Day" using packages 'FactoExtra' and 'FactoMiner' *(57, 58)*. The .csv outputs from the interpolation process in ArcGIS were organized into three separate environmental data sets by their respective depth zones with the boundaries: Shallow (<10 meters), Medium (10-50 meters), and Deep (>50 meters) depth zones as defined by Harris et al. 2014 GSFM (*30*). The two-step PCA and k-means process *(9)* was performed separately for each depth zone. Following removal of SSS due to insufficient data resolution, we proceeded with SST, SST Range, and chl.-α concentration in the analysis. With seasonal variables for SST and chl.-α available, there were a combined total of 11 variables at our disposal for the k-means analysis in each depth zone. Variables were standardized

and converted to z-scores such that 0 was equivalent to the mean and -1 and 1 were equal to the standard deviation of each sample. The z-scored variables were used for evaluating variables and assigning clusters in the PCA and k-means steps, respectively.

A principal components analysis (PCA) was performed to help ensure that selected variables substantially contributed to variance and would lead to the most robust clustering results. Separate PCAs were run for SST and chl.-α in each depth zone, incorporating their respective seasonal and annual averages. We selected the seasonal or annual measurement that contributed the most to variance within the data. SST range was not eligible for this process because only an annual value was available.

Per the PCAs, annual averages contributed more to variance than did seasonal averages for five of the six scenarios run for SST and chl.-α combined across the three depth zones. The one exception was SST for the low depth zone, for which spring SST contributed slightly more to variance across the dataset than annual SST. However, as there was little difference in that case, the analysis proceeded with average annual SST for the low depth zone to maintain consistency across the study.

Proceeding with annual average SST and chl.-α, an additional PCA was conducted to investigate contributions to variance of these variables combined with SST Range for each depth zone. A very strong negative correlation was observed between average annual SST and SST Range; Pearson's Correlation of -0.82, -0.95, and -0.99 for low, medium, and high depth zones respectively. Due to the redundancy of including both SST and SST Range, SST Range was dropped as a variable and the k-means analyses proceeded with average annual SST and chl.-α concentration.

Separate k-means analyses were conducted for each depth zone. A k-means analysis follows two steps such that (1) data points are distributed in Euclidean space, and a preselected number of points (or centroids/cluster centers, *K*) are randomly distributed in that space. Each datapoint is then assigned to the nearest centroid by straight-line Euclidean distance or the minimum square distance criterion. Then (2) the centroid's position is recalculated based on the mean of the datapoints assigned to that centroid/cluster. The process repeats until the value J per the objective function (equation 1) does not significantly change between re-location of cluster centroids, indicating that the clusters are stable *(59)*. The final centroid and datapoint pairing reflect the final cluster assignments for each datapoint. In this algorithm, x_j refers to the position of a given data point in Euclidean space while z_k refers to the position of the given cluster centroid *k*, and u_{kj} = 1 when the datapoint 'belongs' to cluster *k* and u_{kj} = 0 when it does not.

$$(1) \qquad J = \sum_{j=1}^{n} \sum_{k=1}^{K} u_{kj} \left\| x_j - z_k \right\|^2$$

Cluster performance was then measured by observing explained variance for clusters as measured by between the sum of squares (BSS, equation 2) divided by the total sum of squares (TSS, equation 3) where n represents the total number of data points in the cluster, x_k the mean for each cluster, and x_t the sample mean.

$$(1) \qquad BSS = \sum_{k=1}^{K} n(x_k - x_t)^2$$

$$(2) \qquad TSS = \sum (x_j - x_t)^2$$

The ratio of BSS:TSS indicates the percent of variance explained by the clusters, and is an established method for tracking cluster performance *(60)*. We selected a preferred number of clusters that balanced

complexity and cluster performance by observing the marginal increase in the percent of variance explained by this function. We selected 5 clusters for each depth zone at which point additional variance explained began to plateau. The k-means analyses achieved a BSS:TSS of 79.1% for low, 83.6% for medium, and 91.4% for high depth zones (Figure S2). The inclusion of waters beyond the continental shelf as a single habitat provided 16 habitats from which to assess the representation of China's MPA and AGR network. Clusters were validated via pairwise t-tests for explanatory variables to ensure statistically distinct differences between them. For some datapoints, one variable could be extreme enough to define its cluster regardless of other characteristics. For example, habitat D2, characterized by high productivity in deeper waters, included extreme points ranging from the cold Bohai Sea to tropical waters off of Hainan if chlorophyll-α concentrations were high enough (about +2 SD) to statistically offset the influence of temperature on its cluster assignation (Figure S1, S2, and main body Figure 2).

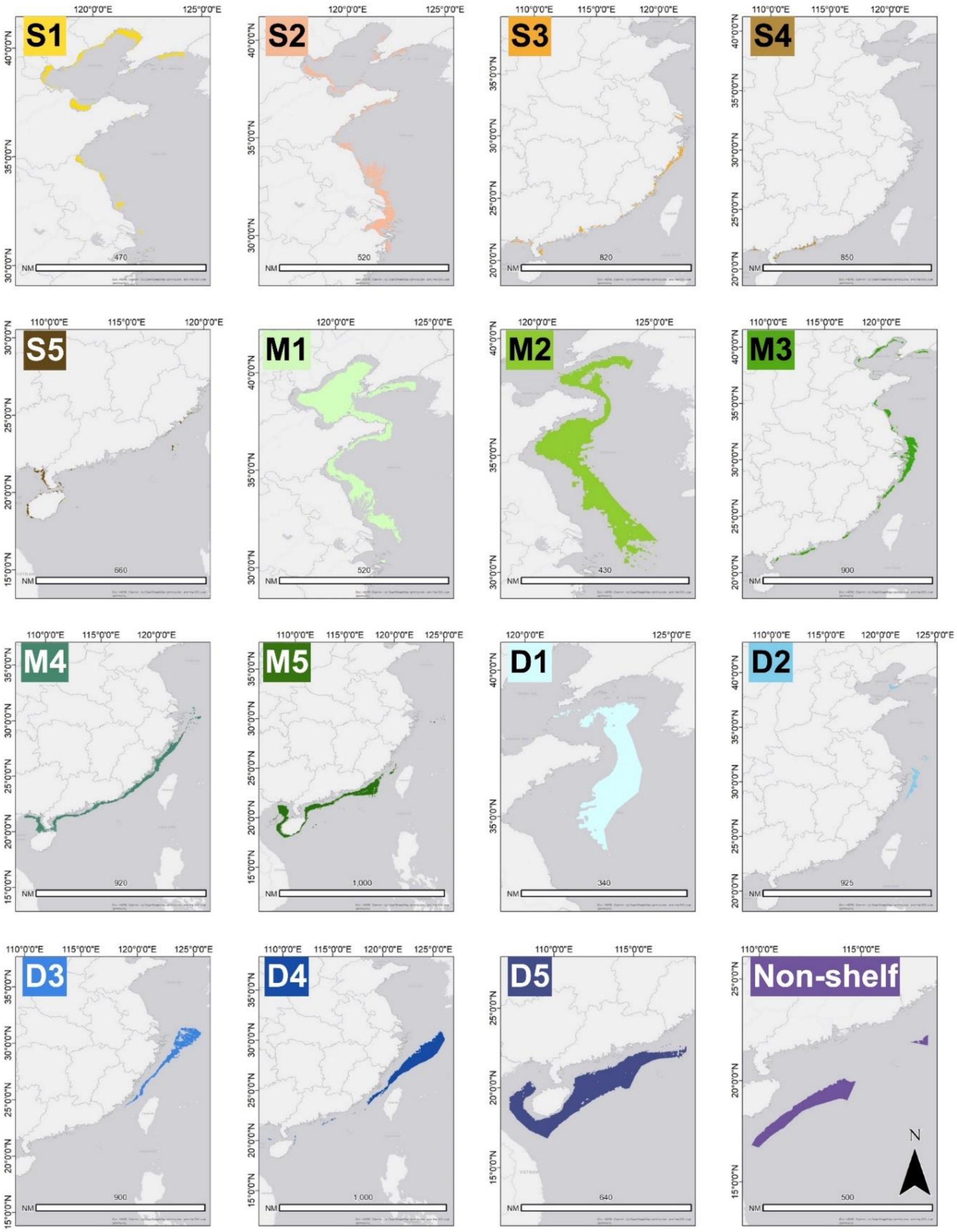

Fig. S1.

The main spatial extent of each of the classified habitats. Mapped using ArcMap 10.7.1 (27), in China Geodetic Coordinate System 2000. Provincial boundaries provided by ArcGIS Online via ArcMap data portal (Table S2).

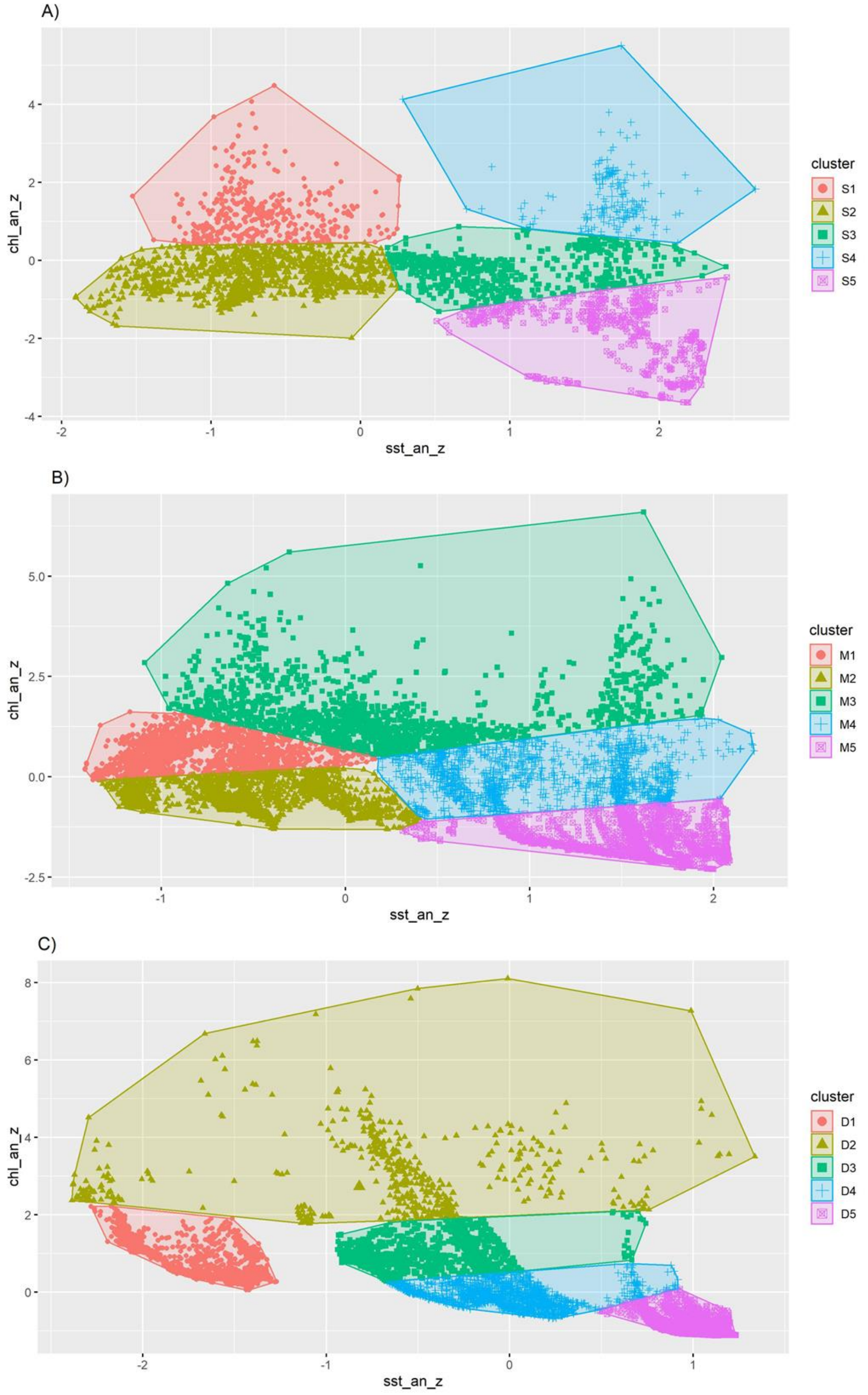

Fig. S2.

Visualization of partitioning results for k-means analysis (k = 5) for clusters representing habitats across the A) shallow (BSS:TSS = 79.1%), B) medium (BSS:TSS = 83.6%), and C) deep (BSS:TSS = 91.4%) depth zones. Final positions on the figure are influenced by annual average SST (sst_an_z) and chl.-α concentrations (chl_an_z) in Euclidean space

**Variable**	Resolution	Year(s) produced	Source
**Chlorophyll-α concentration (chl.-α)**	0.1 degrees	2010 – 2019	Derived from in-situ and Moderate Resolution Imaging Spectroradiometer ocean color measurements
**Sea Surface Temperature (SST)**	0.1 degrees	2010 – 2019	Derived from Advanced Very High Resolution Radiometer Pathfinder Program readings
**Sea Surface Salinity (SSS)**	0.5 degrees	2010 – 2019	Derived from AQUARIUS/SAC-D mission using ocean backscatter

Table S1.

Descriptions of environmental data used in this study from NASA's Earth Observations (NEO) database *(53)*.

**File name**	**Use**	**Last Updated**
World Seafloor Geomorphology *(61)*	Determine shelf and non-shelf boundaries; determine representivity of seafloor features	2019
World Exclusive Economic Zone Boundaries *(62)*	Digitize seaward bound of EEZ polygon	2019
Exclusive Economic Zones of Flanders Marine Institute _2018_ v_10 *(63)*	Digitize near-coast seaward bound of EEZ polygon (fill the gap between the above and below boundaries)	2019
China Country Boundary 2018 *(64)*	Digitize coastal bound of EEZ	2019
Seafloor Bathymetry (meters) – Transparent *(55, 65)*	Get bathymetric datapoints throughout EEZ polygon	2017
China County City District Boundaries 2016 *(66)*	Derive centroids for MPAs via "GIS method"	2019
China Province Boundaries 2018 *(67)*	Visualization	2019

Table S2
Characteristics of the spatial data derived from ArcMap 10.7.1 *(49)* data portal.

REFERENCES AND NOTES

1. L. Cao, R. Naylor, P. Henriksson, D. Leadbitter, M. Metian, M. Troell, W. Zhang, China’s aquaculture and the world’s wild fisheries. *Science* **347**, 133–135 (2015).

2. World Bank (from FAO) World Development Indicators, Capture fisheries production (metric tons); https://data.worldbank.org/indicator/ER.FSH.CAPT.MT?end=2016&start=1960&view=chart&year_high_desc=true).

3. L. Cao, Y. Chen, S. Dong, A. Hanson, B. Huang, D. Leadbitter, D. C. Little, E. K. Pikitch, Y. Qiu, Y. S. De Mitcheson, U. R. Sumaila, M. Williams, G. Xue, Y. Ye, W. Zhang, Y. Zhou, P. Zhuang, R. L. Naylor, Opportunity for marine fisheries reform in China. *Proc. Natl. Acad. Sci. U.S.A.* **114**, 435–442 (2017).

4. D. Tickler, J. J. Meeuwig, M. L. Palomares, D. Pauly, D. Zeller, Far from home: Distance patterns of global fishing fleets. *Sci. Adv.* **4**, eaar3279 (2018).

5. C. Sang-Hun, Defying U.N. Ban, Chinese Ships Pay North Korea to Fish in Its Waters. *New York Times*, p. 10 (2021).

6. I. Urbina, “Fish farming is feeding the globe. what’s the cost for locals?,” *New Yorker* (2021); https://newyorker.com/magazine/2021/03/08/fish-farming-is-feeding-the-globe-whats-the-cost-for-locals.

7. D. Collyns, “Chinese fishing armada plundered waters around Galapagos, data shows,” *Guardian* (2020); https://theguardian.com/environment/2020/sep/17/chinese-fishing-armada-plundered-waters-around-galapagos-data-shows.

8. J. Day, N. Dudley, M. Hockings, G. Holmes, D. Laffoley, S. Stolton, S. Wells, *Guidelines for Applying the IUCN Protected Area Management Categories to Marine Protected Areas*” (International Union for Conservation of Nature and Natural Resources, 2012).

9. J. J. Bohorquez, A. Dvarskas, E. K. Pikitch, Categorizing global MPAs: A cluster analysis approach. *Mar. Policy*. **108**, 103663 (2019).

10. B. C. O’Leary, N. C. Ban, M. Fernandez, A. M. Friedlander, P. García-Borboroglu, Y. Golbuu, P. Guidetti, J. M. Harris, J. P. Hawkins, T. Langlois, D. J. McCauley, E. K. Pikitch, R. H. Richmond, C. M. Roberts, Addressing criticisms of large-scale marine protected areas. *Bioscience* **68**, 359–370 (2018).

11. L. M. Brander, P. van Beukering, L. Nijsten, A. McVittie, C. Baulcomb, F. V. Eppink, J. A. Cado van der Lelij, The global costs and benefits of expanding marine protected areas. *Mar. Policy*. **116,** 103953 (2020).

12. N. Pascal, A. Brathwaite, L. Brander, A. Seidl, M. Philip, E. Clua, Evidence of economic benefits for public investment in MPAs. *Ecosyst. Serv.* **30**, 3–13 (2018).

13. Secretariat of the Convention on Biological Diversity, “Global Biodiversity Outlook 5” (Montreal, 2020).

14. Y. Li, D. L. Fluharty, Marine protected area networks in China: Challenges and prospects. *Mar. Policy* **85**, 8–16 (2017).

15. C. Ma, X. Zhang, W. Chen, G. Zhang, H. Duan, M. Ju, H. Li, Z. Yang, China’s special marine protected area policy: Trade-off between economic development and marine conservation. *Ocean Coast. Manag.* **76**, 1–11 (2013).

16. W. Qiu, B. Wang, P. J. S. Jones, J. C. Axmacher, Challenges in developing China’s marine protected area system. *Mar. Policy* **33**, 599–605 (2009).

17. W. Lu, J. Liu, X. Xiang, W. Song, A. Mcilgorm, A comparison of marine spatial planning approaches in China: Marine functional zoning and the marine ecological red line. *Mar. Policy* **62**, 94–101 (2015).

18. W. Hu, J. Liu, Z. Ma, Y. Wang, D. Zhang, W. Yu, B. Chen, China’s marine protected area system: Evolution, challenges, and new prospects. *Mar. Policy* **115**, 103780 (2020).

19. Y. Li, Y. Ren, Y. Chen, China fortifies marine protection areas against climate change. *Nature* **573**, 346 (2019).

20. W. Zhou, M. Wang, M. Huang, F. Wei, A marine biodiversity plan for China and beyond. *Science* **371**, 685–686 (2021).

21. United Nations Environment Programme, “Strategic Plan for Biodiversity 2011–2020: Further Information Related to the Technical Rationale for the Aichi Biodiversity Targets, Including Potential Indicators and Milestones” (New York, 2011).

22. United Nations Department of Economic and Social Affairs, “The Sustainable Development Goals Report” (New York, 2017), doi:10.18356/3405d09f-en.

23. CBD, TARGET 11 - Technical Rationale extended. *Strategic Plan for Biodiversity 2011-2020* (2010).

24. Convention on Biological Diversity, Quick guide to the Aichi Biodiversity Targets: Protected areas increased and improved (2020); https://cbd.int/doc/strategic-plan/targets/T11-quick-guide-en.pdf.

25. N. J. Gownaris, C. M. Santora, J. B. Davis, E. K. Pikitch, Gaps in protection of important ocean areas: A spatial meta-analysis of ten global mapping initiatives. *Front. Mar. Sci.* **6**, 650 (2019).

26. A. Fischer, D. Bhakta, M. Macmillan-Lawler, P. Harris, Existing global marine protected area network is not representative or comprehensive measured against seafloor geomorphic features and benthic habitats. *Ocean Coast. Manag.* **167**, 176–187 (2019).

27. T. Agardy, J. Claudet, J. C. Day, ‘Dangerous Targets’ revisited: Old dangers in new contexts plague marine protected areas. *Aquat. Conserv. Mar. Freshw. Ecosyst.* **26**, 7–23 (2016).

28. Campaign for Nature, *50 Countries Announce Bold Commitment To Protect At Least 30% of the World's Land and Ocean By 2030* (Campaign for Nature, 2021); https://campaignfornature.org/50-countries-announce-bold-commitment-to-protect-at-least-30-of-the-worlds-land-and-ocean-by-2030.

29. The United Nations, "*United Nations Convention on the Law of the Sea*" (The United Nations, 1982); https://un.org/depts/los/convention_agreements/texts/unclos/unclos_e.pdf.

30. P. T. Harris, M. Macmillan-Lawler, J. Rupp, E. K. Baker, Geomorphology of the oceans. *Mar. Geol.* **352**, 4–24 (2014).

31. Y. Stratoudakis, A. Hilario, D. Abecasis, E. J. Gonçalves, F. Andrade, G. P. Carreira, J. M. S. Gonçalves, L. Freitas, L. Menezes, M. I. Batista, M. Henriques, P. B. Oliveira, P. Oliveira, P. Afonso, P. I. Arriegas, S. Henriques, Environmental representativity in marine protected area networks over large and partly unexplored seascapes. *Glob. Ecol. Conserv.* **17**, e00545 (2019).

32. K. E. Roberts, G. A. Duffy, C. N. Cook, Bio-physical models of marine environments reveal biases in the representation of protected areas. *Aquat. Conserv. Mar. Freshw. Ecosyst.* (2019).

33. E. Pikitch, P. D. Boersma, I. L. Boyd, D. O. Conover, P. Cury, T. Essington, S. S. Heppell, E. D. Houde, M. Mangel, D. Pauly, É. Plagányi, K. Sainsbury, R. S. Steneck, *Little Fish, Big Impact: Managing a Crucial Link in Ocean Food Webs* (Lenfest Ocean Program, 2012).

34. R. R. Reeves, J. Y. Wang, S. Leatherwood, The finless porpoise *Neophocaena phocaenoides* (G. Cuvier, 1829): A Summary of current knowledge and recommendations for conservation action. *Asian Mar. Biol.* **14**, 111–143 (1997).

35. J. Wu, H. Zhang, Y. Pan, D. Krause-Jensen, Z. He, W. Fan, X. Xiao, I. Chung, N. Marbà, O. Serrano, R. B. Rivkin, Y. Zheng, J. Gu, X. Zhang, Z. Zhang, P. Zhao, W. Qiu, G. Chen, C. M. Duarte, Opportunities for blue carbon strategies in China. *Ocean Coast. Manag.* **194**, 105241 (2020).

36. J. Bohorquez, A. Dvarskas, E. K. Pikitch, Filling the data gap - a pressing need for advancing MPA sustainable finance. *Front. Mar. Sci.* **6**, 45 (2019).

37. D. Pauly, D. Zeller, Eds. *Global Atlas of Marine Fisheries: A Critical Appraisal of Catches and Ecosystem Impacts*, (Island Press, 2016).

38. M. Young, M. Carr, Assessment of habitat representation across a network of marine protected areas with implications for the spatial design of monitoring. *PLOS ONE* **10**, e0116200 (2015).

39. A. Zlinszky, H. Heilmeier, H. Balzter, B. Czúcz, N. Pfeifer, Remote sensing and GIS for habitat quality monitoring: New approaches and future research. *Remote Sens. (Basel)* **7**, 7987–7994 (2015).

40. D. A. Gill, M. B. Mascia, G. N. Ahmadia, L. Glew, S. E. Lester, M. Barnes, I. Craigie, E. S. Darling, C. M. Free, J. Geldmann, S. Holst, O. P. Jensen, A. T. White, X. Basurto, L. Coad, R. D. Gates, G. Guannel, P. J. Mumby, H. Thomas, S. Whitmee, S. Woodley, H. E. Fox, Capacity

shortfalls hinder the performance of marine protected areas globally. *Nature* **543**, 665–669 (2017).

41. G. J. Edgar, R. D. Stuart-Smith, T. J. Willis, S. Kininmonth, S. C. Baker, S. Banks, N. S. Barrett, M. A. Becerro, A. T. F. Bernard, J. Berkhout, C. D. Buxton, S. J. Campbell, A. T. Cooper, M. Davey, S. C. Edgar, G. Försterra, D. E. Galván, A. J. Irigoyen, D. J. Kushner, R. Moura, P. E. Parnell, N. T. Shears, G. Soler, E. M. A. Strain, R. J. Thomson, Global conservation outcomes depend on marine protected areas with five key features. *Nature*. **506**, 216–220 (2014).

42. C. Shepherd, L. Hook, China vies with US for lead in global climate diplomacy. *Financial Times* (2021); https://ft.com/content/b243ed83-70c4-4daa-acef-3a28f26e6e8b.

43. Z. Da Ros, A. D. Anno, T. Morato, A. K. Sweetman, M. Carreiro-silva, C. J. Smith, N. Papadopoulou, C. Corinaldesi, S. Bianchelli, C. Gambi, R. Cimino, P. Snelgrove, C. Lee, V. Dover, R. Danovaro, The deep sea: The new frontier for ecological restoration. *Mar. Policy*. **108**, 103642 (2019).

44. K. A. Miller, K. F. Thompson, P. Johnston, D. Santillo, An overview of seabed mining including the current state of development, environmental impacts, and knowledge gaps. **4**, 418 (2019).

45. J. Zeng, *Chinese Marine Protected Areas* (Ocean Press, 2013).

46. "Nationwide protected areas list" (2019); www.gov.cn.

47. "National auatic germplasm reserves area, extent, and subdivisions" (Baidu, 2015), vol. 1–8; wenku.baidu.com.

48. Flanders Marine Institute, Maritime Boundaries Geodatabase: Maritime Boundaries and Exclusive Economic Zones (200NM), version 10. *ArcMap 10.7.1 data portal* (2018).

49. ESRI, ArcGIS Desktop: Release 10.7.1 (2019).

50. P. Cheng, H. Wen, Y. Cheng, H. Wang, *China Geodetic Coordinate System 2000* (Bangkok, Thailand, 2009), doi:10.1007/s11434-009-0342-9.

51. K. W. Yen, H. J. Lu, Y. Chang, M. A. Lee, Using remote-sensing data to detect habitat suitability for yellowfin tuna in the Western and Central Pacific Ocean. *Int. J. Remote Sens.* **33**, 7507–7522 (2012).

52. E. A. Becker, K. A. Forney, M. C. Ferguson, D. G. Foley, R. C. Smith, J. Barlow, J. V. Redfern, Comparing California current cetacean-habitat models developed using in situ and remotely sensed sea surface temperature data. *Mar. Ecol. Prog. Ser.* **413**, 163–183 (2010).

53. NASA, NASA Earth Observations; https://neo.sci.gsfc.nasa.gov/.

54. L. Ouyang, F. Hui, L. Zhu, X. Cheng, B. Cheng, M. Shokr, J. Zhao, M. Ding, T. Zeng, The spatiotemporal patterns of sea ice in the Bohai Sea during the winter seasons of 2000–2016. *Int. J. Digit. Earth*. **12**, 893–909 (2019).

55. J. J. Becker, D. T. Sandwell, W. H. F. Smith, J. Braud, B. Binder, J. Depner, Global Bathymetry and elevation data at 30 arc seconds resolution: SRTM30_PLUS. *Mar. Geod.* **32**, 355–371 (2009).

56. ESRI, IDW. *ArcMap 10.3*; https://desktop.arcgis.com/en/arcmap/10.3/tools/geostatistical-analyst-toolbox/idw.htm.

57. R Core Team, R: A language and environment for statistical computing (2020); https://r-project.org/.

58. S. Le, J. Josse, F. Husson, FactoMineR: An R package for multivariate analysis. *J. Stat. Softw.* **25**, 1–18 (2008).

59. U. Maulik, S. Bandyopadhyay, Performance evaluation of some clustering algorithms and validity indices. *IEEE Trans. Pattern Anal. Mach. Intell.* **24**, 1650–1654 (2002).

60. M. J. Crawley, *The R Book* (John Wiley & Sons Ltd., ed. 2, 2013).

61. Esri ArcGIS Online, World Seafloor Geomorphology (Esri ArcGIS Online, 2019); https://oceans1.arcgis.com/arcgis/rest/services/World_Seafloor_Geomorphology/MapServer.

62. Esri ArcGIS Online, World Exclusive Economic Zone Boundaries (Esri ArcGIS Online, 2019).

63. Esri ArcGIS Online, Exclusive Economic Zones of Flanders Marine Institute_2018_v_10 (2019); https://services3.arcgis.com/2LJ6Q6yCIIUOP8qx/arcgis/rest/services/Exclusive_Economic_Zones_of_Flanders_Marine_Institute__2018__v_10/FeatureServer.

64. Esri ArcGIS Online, China Country Boundary 2018 (2018); https://services.arcgis.com/P3ePLMYs2RVChkJx/arcgis/rest/services/CHN_Boundaries_2018/FeatureServer.

65. Esri ArcGIS Online, Seafloor Bathymetry (meters) - Transparent (Esri ArcGIS Online, 2017); https://oceans2.arcgis.com/arcgis/rest/services/Seafloor_Bathymetry/ImageServer.

66. Esri ArcGIS Online, China County City District Boundaries (Esri ArcGIS Online, 2016); https://services.arcgis.com/P3ePLMYs2RVChkJx/arcgis/rest/services/CHN_Boundaries_2016/FeatureServer.

67. Esri ArcGIS Online, China Province Boundaries 2018 (Esri ArcGIS Online, 2018); https://services.arcgis.com/P3ePLMYs2RVChkJx/arcgis/rest/services/CHN_Boundaries_2018/FeatureServer.
